# Using Step Trackers Among Older People Receiving Aged Care Services Is Feasible and Acceptable: A Mixed-Methods Study

**DOI:** 10.3390/healthcare14010086

**Published:** 2025-12-30

**Authors:** Rik Dawson, Judy Kay, Lauren Cameron, Bernard Bucalon, Catherine Sherrington, Abby Haynes

**Affiliations:** 1 Institute for Musculoskeletal Health, The University of Sydney, Sydney Local Health District, Gadigal Country, Sydney, NSW 2000, Australiaabby.haynes@sydney.edu.au (A.H.); 2 Human-Centred Computing, School of Computer Science, The University of Sydney, Gadigal Country, Sydney, NSW 2000, Australia

**Keywords:** fall prevention, wearable activity trackers, Fitbits, physiotherapy, digital health, physical activity, older people, aged care

## Abstract

**Highlights:**

**What are the main findings?**

- Step tracking was feasible and acceptable for older adults receiving aged care, with strong engagement and a clear preference for Fitbit devices over phone or website interfaces.
- Wrist-worn trackers increased physical activity awareness and motivation, supported by reminders, rewards, and social sharing.

**What are the implications of the main findings?**

- Digital activity monitoring for older people is likely to benefit from simple, user-friendly designs that provide quick and accessible feedback.
- Wearable trackers may offer a practical, scalable way to enhance motivation, support restorative care, and strengthen social connectedness in aged care settings.

**Abstract:**

**Background:** Maintaining physical activity (PA) is vital for older people, particularly those with frailty and mobility limitations. Wearable activity trackers and digital feedback tools show promise for encouraging PA, but their feasibility and acceptability in aged care remain underexplored. This study evaluated the feasibility and acceptability of using wearable and mobile devices for step tracking and examined the usability of three interfaces (Fitbit, mobile app, and website) for reviewing PA progress in aged care. **Methods:** This is a user experience and feasibility study that does not involve objective physical activity quantification or device performance analysis. It is a mixed-methods feasibility study conducted with 14 participants aged ≥65 years from residential and community aged care services in metropolitan and regional New South Wales, Australia. Participants used a Fitbit Inspire 3 linked to a study website and a mobile phone step-counting app to monitor their steps across the three interfaces for four weeks. Feasibility was evaluated through enrolment and retention, and acceptability through a facilitator-led survey. Quantitative items on usability, comfort, motivation and device preference were summarised descriptively; open-ended responses were analysed thematically to identify user experiences, benefits, and barriers. **Results:** Step tracking was feasible, with 82% enrolment and 93% retention. Participants preferred the Fitbit over the mobile phone or website due to its ease of use, visibility and more enjoyable experience. Step tracking increased awareness of PA and supported confidence to move more. Participants valued reminders, rewards and opportunities for social sharing. Reported barriers included illness, usability challenges and occasional technical issues. **Conclusions:** Wearable step trackers show promise for supporting PA among older people receiving aged care. Despite the small sample and short follow-up, strong acceptability signals suggest that simple digital tools could enhance the reach and sustainability of mobility-promoting interventions into aged care systems. Future studies should examine long-term adherence, usability across diverse mobility and cognitive needs, and conditions for successful scale-up.

## 1. Introduction

Physical activity (PA) is essential for healthy ageing, supporting functional independence, mental well-being and quality of life among older people [1]. Yet advancing age is consistently associated with declines in activity and increases in sedentary behaviour [2], particularly among those with frailty and multiple comorbidities receiving aged care services [3]. In this population, low PA levels are strongly linked to a higher risk of falls [4], functional decline, and premature institutionalisation [5]. With populations ageing rapidly worldwide, scalable and cost-effective solutions to support PA and reduce fall risks are urgently required.

Digital activity monitoring devices, including step tracking wearables (e.g., Fitbits) and smartphone applications, are promoted to support behaviour change through self-monitoring, feedback, and goal setting [6]. Wearables provide detailed activity metrics [7] and have been shown to increase activity levels in community-dwelling older people by enhancing PA awareness and motivation [8]. However, evidence for their feasibility and usability among frail older adults receiving aged care services remains limited, particularly given common challenges such as mobility impairment, cognitive decline and variable digital literacy [9].

Although device preferences vary, existing research offers mixed findings and largely excludes frail older adults. There is insufficient evidence to determine which interface, wearable, smartphone app or website, is most usable, acceptable or practical in aged care contexts [10]. The present study builds on findings from the TOP-UP trial, a hybrid effectiveness–implementation randomised controlled trial of a programme comprising physiotherapist-led telehealth, local support and exercise videos in aged care. The trial demonstrated a 38% reduction in fall risk, clinically meaningful improvements in mobility [11] and strong acceptability among participants and aged care staff [12]. However, several interviewees suggested that exercise engagement could diminish once regular support was no longer available. This reinforces the value of simple behavioural supports such as feedback, goal setting and self-monitoring to help sustain PA participation [12].

While PA feedback is often promoted as a behaviour change tool, there is limited evidence on whether such approaches are feasible or acceptable for frail older people [9]. This mixed-methods study addresses that evidence gap by assessing the feasibility and acceptability of using wearable and mobile devices for step tracking among older people receiving aged care services. It also explored user experience across three interfaces, a wearable device, smartphone app, and linked website, to inform the development of a more comprehensive digital health platform to promote PA and engagement in aged care.

## 2. Method

This feasibility study was approved by the Sydney Local Health District Human Research Ethics Committee, Concord (approval number CH62/6/2024-019, approved 18 March 2024). All participants provided written informed consent prior to enrolment. The study adhered to the principles of the Declaration of Helsinki. Recruitment occurred between August and September 2024.

### 2.1. Eligibility Criteria

Eligible participants were aged 65 years and older, either living independently in the community or residing in a residential aged care facility (nursing home/long-term care home). All participants were recipients of Australian Government funded aged care services, which provide subsidised support for daily living, health care, and personal assistance for older people. Inclusion criteria required ownership of a compatible smartphone (Android or iOS) and willingness to use it during the study, as well as sufficient physical and cognitive ability to operate the three devices. The latter was assessed informally by the recruiting physiotherapist (RD) through brief functional checks. Exclusion criteria included severe cognitive impairment (defined as a score of ≤10 on the Modified Telephone Interview for Cognitive Status) [13] and an inability to walk 50 metres independently with or without a walking aid.

### 2.2. Recruitment and Consent

Participants were recruited in collaboration with three aged care service provider partners in Sydney, Newcastle, and regional New South Wales, Australia. Service users received an information sheet outlining the study, followed by a phone call from a researcher (RD) to explain the aims and procedures and address any questions. Prospective participants could decline, request additional time to consider (with a follow-up call within one week), or provide immediate written consent. Participation was voluntary, and individuals were informed that they could withdraw from the study at any time without penalty or consequence, and that doing so would not affect their ongoing relationship with their aged care service provider.

### 2.3. Step Tracking Intervention

**Figure 1:**
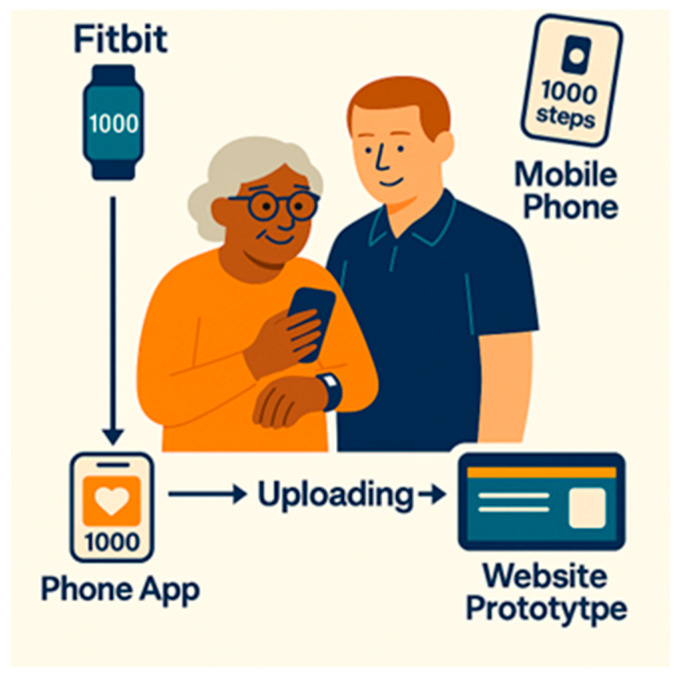
Step tracker programme components and data flow.

Participants used two step tracking tools during the four-week trial: a *Fitbit Inspire 3* and a smartphone step-counting app. The Fitbit synchronised with a secure study app that exported deidentified step data to the prototype TOP UP website. Smartphone data were captured through a free pedometer app (*Pedometer Step Counting*) and viewed directly on the phone. Figure 1 shows how participants accessed their progress across the three interfaces. The prototype website enabled testing of how activity monitoring could link with the TOP-UP platform and whether participants could compare step-count information across interfaces.

Participants were asked to review their step counts daily on all three interfaces to assess usability and data display preferences. Website access occurred via a loaned iPad or participants’ own tablets. Researchers provided in-person training, with additional help available from local support workers and telephone support from the study team. De-identified step data and user feedback were shared with the website developer to test data flows, identify integration barriers and refine the user experience for future trials. Participants kept the Fitbit after completing the study to support ongoing activity monitoring.

### 2.4. Data Collection

Feasibility was assessed through enrolment and retention rates, attrition reasons and the ability to collect step-count data. Participants used the Fitbit, phone app and website simultaneously, and the survey and interview guides were structured to prompt interface-specific feedback. Participants were asked about each platform separately, enabling clear differentiation of their experiences across the three interfaces. Acceptability was explored using a 30 min semi-structured, researcher-facilitated survey (face to face or via Zoom; Appendix A). The survey was researcher-developed for this feasibility study, informed by existing usability constructs but not adapted from a validated instrument. Surveys included Likert-scale items assessing usability, comfort and motivation, as well as open-ended questions exploring perceptions, preferences (Fitbit vs. mobile phone vs. website) and barriers to sustained use. Adverse events, including falls, were monitored throughout. Two researchers conducted data collection: RD, a male postdoctoral physiotherapist with extensive aged-care experience, and LC, a female user experience researcher with qualitative research expertise. A sample of 14 participants is consistent with feasibility study guidance and sufficient to identify major acceptability and usability patterns, achieve data saturation and thematic sufficiency, and inform procedural refinements, noting that the aim was not to achieve statistical power [14].

### 2.5. Analysis

Quantitative survey data was analysed descriptively using median, interquartile ranges (IQRs) and ranges to summarise usability, comfort, and engagement. Qualitative responses were analysed using inductive content analysis [15]. RD and LC coded responses into categories (e.g., ease of use, visibility, charging requirements) and developed themes supported by illustrative quotes. Emergent findings were presented to the wider research team, who acted as “critical friends” [16]. This is a user experience and feasibility study that did not involve objective physical activity quantification or device performance analysis. No statistical analysis of step-count data was undertaken, as objective metrics such as wear-time, data completeness, sync performance and raw step-count outputs were not collected or retained. This feasibility study focused specifically on acceptability, usability and participant experience rather than behavioural outcomes or device performance, and the analysis approach was aligned with these predefined aims.

## 3. Results

Fourteen aged care service users were enrolled in the four-week study, representing 82% of those approached (14/17), and 93% (13/14) completed the study. One participant withdrew in week 3 due to deteriorating health but was available for the follow-up survey. The cohort comprised six men and eight women, with seven participants residing in residential aged care and seven receiving home-based services. The median age was 83 years (range 68–94). Five participants (36%) used a walking frame for safe mobility, and five (36%) had a diagnosis of mild cognitive impairment. No adverse events were recorded during the study period.

Participants generally reported positive experiences with step tracking. Most indicated that step monitoring increased their PA awareness and supported daily motivation to be active. Survey findings are presented in Table 1. Device descriptive preference was clear: twelve participants (86%) favoured Fitbit devices, citing ease of use, comfort, and less frequent charging. Two participants (14%) expressed no device preference. Fitbits consistently received higher usability and enjoyment scores (median 5, IQR 5–5, range 3–5), whereas mobile phones were rated lower (median 3, IQR 2–4, range 1–4), reflecting the greater effort required for charging, carrying, and operation. All but one participant intended to continue step tracking beyond the study.

### Content Analysis of Open-Ended Survey Responses

The five subsections below represent themes derived from qualitative content analysis of open-ended survey responses. (Supplementary File S1). These themes mapped directly to the study aims: the potential of step tracking to motivate engagement, the barriers and enablers to its use, and participants’ device preferences.

1. “Doing something positive”: Finding purpose in step tracking

Participants consistently reported that step tracking gave them a sense of purpose and achievement. They indicated that monitoring progress fostered motivation and pride and encouraged goal setting. Participant 13 reflected, “*It was great to see how much walking I got in, made me feel really great that I was doing something positive for my life*.” For others, step counts reframed walking as pleasurable and a meaningful achievement. Participant 2 noted, “*Checking my steps made my walking feel not like exercise, made it more enjoyable*.”

2. “A little push helps”: Gamification and personal challenge

Interactive features such as reminders, rewards and milestone notifications were reported by participants as motivating. Participants described striving for daily milestones or turning step tracking into a personal challenge. Participant 8 explained, “*My Fitbit motivates me with rewards and messages to get walking, helping me reach 6000 steps daily. This is a big improvement from where I started at 2000 steps*.” Gamified elements and feedback loops appeared to provide steady encouragement and reinforce positive behaviour change.

3. “Sharing steps, sharing stories”: Building social connection

Beyond individual motivation, participants described how step tracking *created opportunities* for social interaction and encouragement. Some used their devices to share progress and engage in gentle competition with peers and family members. Participant 3 noted, “*I like to compare my steps with a friend at the nursing home to see who has done more,”* while Participant 12 shared, “*I liked to show my daughter how I was doing, and I was really pleased that when I was on holidays I did more than 10,000 steps*.” These examples suggest that step tracking may *enhance existing relationships* and make PA feel more shared and socially engaging for some participants.

4. “Not always easy, but worth it”: Barriers and support

Participants described a range of health and practical barriers that limited step tracking at times. Illness, pain, fatigue and medical appointments disrupted routines—as Participant 1 explained, “*During the trial I was unwell and didn’t really have the energy to track my steps. Now I am feeling better and plan to start walking more*.” Others noted challenges with technology and design, including small font sizes, difficulty fastening the device, and occasional syncing or Wi-Fi issues. Participant 2 commented, “*You need to have larger numbers on the Fitbit and the website to make it easier for older people like me to read.*”

Practical help was often key to overcoming these issues. Participant 5 said, “*The Fitbit is a bit difficult to put on, and I had to rely on the staff to put it on and sometimes they forgot*,” while Participant 6 shared, “*Once the Fitbit didn’t work, I had to get my grandson to fix it*.” Despite these challenges, participants described satisfaction and pride when they managed to persist. As Participant 13 reflected, “*I loved using the Fitbit… I got over 10,000 steps one day*.” Together, these accounts suggest that step tracking is *feasible and rewarding* for older people when adequate technical and personal support is available.

5. “Fitbit are easier to use”: Device preferences and usability

Fitbits were preferred over the smartphone application and the website interface, with participants valuing the immediacy of data on their wrist, continuous tracking, and the reduced need to remember to carry or charge a phone or open a website. Participant 7 summarised, “*I found it easier to just tap the Fitbit to see my step count than getting my iPad or mobile phone out of my pocket*.” Although smartphones can display step counts through downloaded apps, in this study step data were also configured to flow into the TOP-UP website to test how activity monitoring might be integrated into the digital platform. While we anticipated that enhanced visual displays and additional graphs on the website might encourage participants to engage with this interface, this was not the case. Accessing data via the website on a tablet or phone was often described as cumbersome or easy to forget, as it required logging in and navigating the site. Participant 13 admitted, “*I am terrible with mobiles and computers, and I didn’t check the website at all. It just seemed like a bother to sit down and open up the website*.” Similarly, Participant 6 noted, “*It was easier to remember to put my Fitbit on. I didn’t check the website once because I kept forgetting to*.” Overall, Fitbits were regarded as superior for supporting positive behaviour change. Participant 4 shared, “*I found the Fitbit easy to use and wore it all the time. I liked keeping track of my steps and checked my Fitbit multiple times a day—it pushed me to do more steps and gave me courage to do more*.”

## 4. Discussion

This study demonstrated that routine use of PA trackers is both feasible and acceptable for older people receiving aged care services. High enrolment (82%) and retention (93%) rates, combined with participants’ intention to continue step tracking, support the practicality of these devices in this setting. Participants valued the motivational features and convenience of wrist-worn trackers compared with mobile phones or website interfaces. Step tracking enhanced awareness of PA, encouraged goal setting and progress monitoring, and fostered positive behaviour change.

Our findings align with research showing that digital activity-monitoring devices can help increase PA, including among independently living older people [17]. Systematic reviews confirm that activity trackers and smartphone apps, especially those using feedback, gamification and personalisation, effectively increase step counts across adult populations [7,8,17], though frail or multimorbid older people receiving aged care services are not specifically represented. In our study, participants showed a clear preference for the wrist-worn Fitbit to track their step count, reflected in higher usability ratings (median 5, IQR 5–5) and qualitative feedback describing it as easier to view, simpler to operate and less burdensome than the phone-based interface. The visible, at-a-glance display enabled quick checking of step counts without needing to unlock a phone or navigate an app [18]. Consistent with the broader evidence base, our results suggest that step tracking devices increased awareness of PA and supported motivation to engage with structured programmes, addressing an important evidence gap in this population.

Participants also described individual and interpersonal benefits. Many shared their step-count results with peers or family members, transforming self-monitoring into a socially rewarding and motivating experience. This aligns with evidence that social interaction and shared goals can reduce loneliness and improve well-being among older people [5,7,19,20]. Step trackers may therefore support both behaviour change and social connectedness in aged-care settings.

Barriers to engagement and device adherence were also observed. Health challenges such as illness, fatigue and pain occasionally limited participation, while usability issues, including small font sizes, difficulty reading or fastening devices and technical glitches, reduced confidence and sometimes hindered continuity. Support from staff and family members often enabled participants to persist, reinforcing the importance of providing practical and social assistance during digital health adoption.

Website engagement was particularly limited. Participants found the log-in processes cumbersome compared with the immediacy of viewing steps on the Fitbit. Testing three platforms simultaneously (Fitbit, smartphone app and website) provided useful comparative insights but may also have complicated user experience. Although we anticipated that enhanced graphical displays and additional feedback features might increase engagement, this was not observed. These findings suggest that a website must offer substantially added value, such as larger text, clearer trends or simplified dashboards, to motivate regular use among older adults, particularly those with lower digital literacy.

Despite these challenges, participants demonstrated adaptability and reported pride in mastering new technology. This finding supports broader evidence that simplicity, readability and human support are critical enablers of digital health engagement [9,21]. Support workers and family members therefore play a dual role, providing technical assistance while strengthening social connections.

A strength of this study was its relationship-based approach to engaging participants as partners. Conversational, iterative data collection fostered trust and openness, positioning participants as partners in the research process and generating qualitative insights that complemented survey data. This mixed-methods design enabled a nuanced understanding of contextual factors shaping feasibility and acceptability, reinforcing the value of integrating qualitative approaches into feasibility trials [22,23].

Several limitations should be acknowledged. The trial lasted only four weeks, which restricts conclusions about longer-term feasibility. The small sample size (n = 14) of older people from three aged care providers, and the exclusion of those with severe cognitive or physical disability, limits generalisability. Participants were likely more motivated than the broader aged care population. Objective metrics such as wear time, data completeness and step-count outputs were not collected, as the study focused on feasibility, acceptability and participant experience rather than behavioural outcomes. The study also did not evaluate the accuracy of the Fitbit device or compare step counts with calibrated or research-grade measures. Accuracy may be reduced for people with slower gait speeds or those who use walking aids, and future trials should include formal validation procedures for these groups. Future studies are also needed to assess the effect of step tracking on physical activity levels. Finally, although adverse events were monitored, the limited sample size and short timeframe prevent firm conclusions about safety or risk.

## 5. Conclusions

This feasibility study indicates that wearable and mobile devices are feasible and acceptable for step tracking among older people receiving aged care services. Participants generally preferred the wrist-worn Fitbit, and the website showed early promise for integrating activity data into digital care pathways. However, engagement was shaped by digital literacy, health fluctuations and available support, highlighting the need for caution when implementing digital tools in aged care settings. Future studies should assess long-term adherence, compare interface types and evaluate device accuracy, with larger trials needed to examine behavioural outcomes, safety and scalability.

## Figures and Tables

**Table 1:** Survey ratings of usability and acceptability of step tracking devices (n = 14).

Survey Question	Median	IQR (Q1–Q3)	Range
1. It was easy to use the Fitbit to track my daily step count	5	5–5	3–5 *
2. I liked using the Fitbit to track my steps	5	5–5	3–5 *
3. It was easy to use the mobile phone to track my daily step count	3	2–4	1–5
4. I liked using my mobile phone to track my steps	3	2–4	1–4
5. I liked being able to see my step count	5	5–5	3–5
6. Tracking my steps made me more aware of being physically active	5	4–5	3–5
7. I will continue to track my steps after this study ends	5	5–5	3–5 *

* indicates questions where just one participant had a rating of 3 and every other participant had 5.

## Data Availability

The data sets generated during this study are not publicly available because of confidentiality promised to the participants as part of the informed consent process. However, deidentified data sets are available from the corresponding author upon reasonable request.

## Supplementary Materials

The following supporting information can be downloaded at: https://www.mdpi.com/article/10.3390/healthcare14010086/s1, Supplementary File S1: Thematic Quotes from Step-Tracker Acceptability Study.

## Appendix A. Testing Devices for Tracking Step Counts: Survey Questions

Thank you again for participating in this part of the TOP-UP study. We’re going to discuss your experiences using a Fitbit and mobile phone to track your steps over the last four weeks. These questions will focus on how you used both these devices and how you felt about using them.				
**1. Let’s start with a really broad question: What was it like to use a Fitbit and mobile phone to track your steps?**				
**2. Was it hard to remember to put the Fitbit on each day or to carry your phone when you were moving around?** [if they mention forgetting it sometimes, follow up re how often]				
**3. We’d like to know which of the devices you liked best for tracking your steps: the Fitbit or your mobile phone?** [Talk about their experience first then ask the participants how they would rate each device on this scale]:				

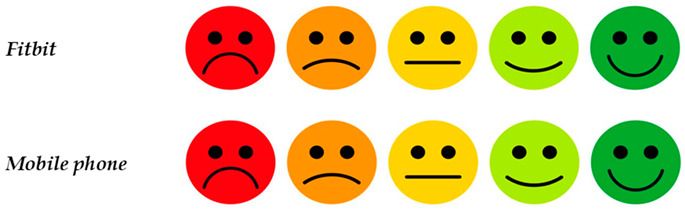

[If one is preferred] **Why was that device preferable?**				
**4. Were there any difficulties or concerns about tracking your steps? If you could change one thing about the Fitbit or about using your phone to track steps, what would it be?**				
**5. What was it like to see the amount of steps you’d done on the iPad? How did it feel?**				
**6. Could we improve the way information about your steps was shown on the website?**				
**7. Do you think that tracking your steps affected the amount of walking or other exercise you did?**				
**I have a few more questions. I’ll read out each statement, and I’d like you to tell me how much you agree or disagree with it. The first one is... ^1^** **:**				
**8. It was easy to use the Fitbit to track my daily step count**				
Strongly disagree	Somewhat disagree	Neither agree nor disagree	Somewhat Agree	Strongly Agree
1	2	3	4	5
**9. I liked using the Fitbit to track my steps**				
Strongly disagree	Somewhat disagree	Neither agree nor disagree	Somewhat Agree	Strongly Agree
1	2	3	4	5
**10. It was easy to use the mobile phone to track my daily step count**				
Strongly disagree	Somewhat disagree	Neither agree nor disagree	Somewhat Agree	Strongly Agree
1	2	3	4	5
**11. I liked using my mobile phone to track my steps**				
Strongly disagree	Somewhat disagree	Neither agree nor disagree	Somewhat Agree	Strongly Agree
1	2	3	4	5
**12. I liked being able to see my step count**				
Strongly disagree	Somewhat disagree	Neither agree nor disagree	Somewhat Agree	Strongly Agree
1	2	3	4	5
**13. Tracking my steps made me more aware of being physically active**				
Strongly disagree	Somewhat disagree	Neither agree nor disagree	Somewhat Agree	Strongly Agree
1	2	3	4	5
**14. Tracking my steps motivated me to walk or exercise more**				
Strongly disagree	Somewhat disagree	Neither agree nor disagree	Somewhat Agree	Strongly Agree
1	2	3	4	5
**15. I will continue to track my steps after this study ends**				
Strongly disagree	Somewhat disagree	Neither agree nor disagree	Somewhat Agree	Strongly Agree
1	2	3	4	5
**16. Finally, is there anything else you can tell us that might help us improve how we engage people in the TOP-UP program?**				
Thank you so much for sharing your views and experiences. It’s really helpful for us to have your input.				
^1^ Explain/negotiate the response options as they answer each question to ensure it’s the best fit for them. Only one whole number can be selected (e.g., 3.5 or 3 to 4 are not acceptable).				
