# Supplementary material for: Using Step Trackers Among Older People Receiving Aged Care Services Is Feasible and Acceptable: A Mixed-Methods Study
Source: Healthcare (Basel). 2025 Dec 30;14(1):86. doi: 10.3390/healthcare14010086 (PMC12785936; doi:10.3390/healthcare14010086)
Supplement: Supplementary file 1 [file healthcare-14-00086-s001.zip › healthcare-4030836-supplementary.pdf]

Supplementary Material File S1: Thematic Quotes from Step Tracker Acceptability Study

1. " Doing something positive": Finding purpose in step tracking

Tracking steps provided motivation for physical activity and inspired users to set and achieve goals.

- 13: It was great to see how much walking I got in, made me feel really great that I was doing something positive for my life.
- 2: "Checking my steps made my walking feel not like exercise, made it more enjoyable."
- 4: "Keeping track of my steps encouraged me to do more and pushed me to go over my goal I set with my physiotherapist."
- 5: "I enjoy seeing how many steps I have done. I feel proud of myself. I can't wait to walk around the shops and see how many steps I do."
- 6: "The Fitbit helps me focus on my walking and got me into better habits."
- 7: "I have always done a lot of walking but I reckon I am doing more now."
- 8: "My Fitbit motivates me with rewards and messages to get walking, helping me reach 6,000 steps daily."
- 12: "Measuring my steps with the Fitbit has made me think that I needed to do more balance exercises."
- 12: "I found the Fitbit easy to use, I liked how it messaged me to charge it. I was even at the movies and it buzzed to tell me it was time to do some more walking."

2. "A little push helps": Gamification and personal challenge

The use of Fitbit's reminders, rewards and milestones features created an engaging and motivating experience for participants. Gamification elements, such as virtual rewards, congratulatory messages, social comparison and friendly competition, encouraged them to increase their step count and sustain their activity levels.

- 3: "I find tracking my steps really motivating, I like to compare my steps with a friend at the nursing home to see who has done more."
- 4: "I really like the prizes FitBit gave me when I achieved certain step milestones – it was very encouraging."
- 5: "I think the reminders that FitBit send you really pushed me to do more. I need the push."
- 7: "I thought the FitBit was really good, initially I didn't think I would use it, but I did, I have been averaging 5000-8000 steps per day—and yesterday I got a laugh when the Fitbit congratulated me on doing 10 000 steps."
- 8: "My Fitbit motivates me with rewards and messages to get walking, helping me reach 6,000 steps daily. This is a big improvement from where I started at 2000 steps. I enjoy comparing my steps with family and friends, often walking more and turning it into a fun competition."

3. "Sharing steps, sharing stories": Building social connection

Participants noted that health issues and a lack of energy sometimes hindered their ability to engage with step tracking.

- 3: "I like to compare my steps with a friend at the nursing home to see who has done more."
- 6: "When I was in pain, I didn't feel like doing a planned walk. Now I am feeling better, it makes me want to do more walking."
- 9: "My big problem is that I didn't have enough time to do more walking due to all my medical appointments."
- 12: "I liked to show my daughter how I was doing, and I was really please that when I was on holidays I did more than 10 000 steps. I made me think I could do more walking at home."
- 14: "Since I have been in a nursing home and not well, I don't have the energy to motivate myself as much as I did when I was living at home."

4. "Not always easy, but worth it": Barriers and support

Technical challenges with Wi-Fi, charging, and device functionality limited engagement and required external help. Older adults faced difficulties with the Fitbit interface, charging, and putting the device on, often needing external assistance to use the step tracker devices.

- 1: "During the trial I was unwell and found that I didn't really have the energy to track my steps. Now I am feeling better and plan to start walking more"
- 1: "It was difficult to read the step count on the Fitbit, the writing was too small."
- 1: "I found that the Fitbit lost its charge quickly and I needed help charging it but I did wear it every day."
- 2: "I had some troubles with Wi-Fi which meant that some days my FitBit wasn't working."
- 2: "I think you need to have larger numbers on the FitBit and the website to make it easier for older people like me to read it."
- 5: "The FitBit is a bit difficult to put on and I had to rely on the staff to put it on and sometimes they forgot."
- 6: "Once the Fitbit didn't work, I had to get my grandson to fix it."
- 9: "I would like a bigger watch face on the FitBit so I could see it more easily for my ageing eyes."
- 13: "I loved using the Fitbit, it was really useful to know how many steps I have done, I got over 10 000 steps one day."
- 13: "I did have some problem with the Fitbit measuring the steps, and I think it may be due to the inconsistent Wi-Fi."

5. Device Preference and Ease of Use

Fitbits were generally preferred over other devices like phones or websites for step tracking due to convenience and ease of access.

- 3: “I found it much easier looking at my steps app on my mobile or FitBit watch to track my steps than the website as the writing was too small, and it was a bit of a hassle to log on.”
- 4: “I found the FitBit easy to use and I wore it all the time. I like to keep track of my steps and I checked my FitBit multiple times a day, it pushed me to do more steps – gave me courage to do more.”
- 6**:** “It took me some time to get in the habit to carry my mobile phone all the time, it was easier to remember to put my Fitbit on. I didn’t check the website once because I kept forgetting to.”
- 7: “I found it easier to just tap the Fitbit to see my step count than getting my iPad or mobile phone out of my pocket.”
- 8: “The Fitbit is a lot easier; it starts recording from midnight, while the phone only counts your steps when you remember to carry it.”
- 9: “I often forgot to carry my mobile phone around with me inside the house, and I found the Fitbit easier.”
- 13: “I am terrible with mobiles and computers and I didn’t check the website all. It just seemed like a bother to sit down and open up the website.
